# Representativeness of personality and involvement preferences in a web-based survey on healthcare decision-making

**DOI:** 10.1186/s12913-020-05717-1

**Published:** 2020-09-10

**Authors:** Søren Birkeland, Thea Linkhorst, Anders Haakonsson, Michael John Barry, Sören Möller

**Affiliations:** 1 Department of Clinical Research, University of Southern Denmark and Open Patient data Explorative Network, Odense University Hospital, J. B. Winsløws Vej 9 a, 3. floor, DK-5000 Odense C, Denmark; 2 MGH Division of General Internal Medicine & Harvard Medical School, Boston, USA

**Keywords:** Research methodology, Internet-based survey, Representativeness, Generalizability, Personality, Decision making, Bioethics, Medical law, Cancer

## Abstract

**Background:**

Obtaining a sample that is representative of the group of interest is of utmost importance in questionnaire studies. In a survey using a state authorized web-portal for citizen communication with authorities, we wanted to investigate the view of adult men on patient involvement in health care decision-making regarding Prostate-Specific Antigen test for prostatic cancer. *In this paper, we report on sample characteristics and representativeness of our sample in terms of personality and baseline involvement preferences*.

**Methods:**

We compared personality profiles (BFI-10) and baseline healthcare decision-making preferences (CPS) in our sample (*n* = 6756) to internationally available datasets. Pooled data from a) US, UK, Canada, Australia, and New Zealand (*n* = 1512), b) Germany, Netherlands, Switzerland, and Belgium (*n* = 1136), and c) Norway, Sweden, Finland, and Denmark (*n* = 1313) were used for BFI-10 comparisons. Regarding CPS, we compared our sample with three previous datasets relating to decision-making in cancer (*n* = 425, 387, and 199).

**Results:**

Although statistically significant differences particularly appeared in large dataset comparisons, sample BFI-10 and CPS profiles mostly were within the range of those previously reported. Similarity was greatest in BFI-10 comparisons with group a) where no statistically significant difference could be established in factors ‘agreeableness’ and ‘neuroticism’ (*p* = .095 and .578, respectively).

**Conclusion:**

Despite some variation, our sample displays personality and baseline preference profiles that are generally similar to those described in previous international studies. For example, this was the case with the BFI-10 ‘agreeableness’ measure (incl. trust and fault-finding items), an important factor in healthcare decision-making.

## Background

Communicating with patients plays an important role in health care. In this regard, since patients must bear the consequences of health care decisions, a mandatory role for patients is increasingly being recommended [1, 2]. Research has suggested that problematic communication and poorly delivered information is often a major reason when patients decide to initiate a malpractice action [3, 4]. Nonetheless, we still have little knowledge about whether greater patient involvement in health care decision-making improves satisfaction and reduces a patient’s likelihood to initiate a malpractice complaint [2, 5].

In order to study these potential relationships, we conducted a large national survey with hypothetical vignettes illustrating various levels of patient participation in decision-making together with different outcomes. Development of the survey, its core structure, and examples of survey elements have been previously described [2]. Following completion of the survey, sample representativeness came into question [6, 7]. The representativeness of a sample can be defined in terms of its external validity in relation to the target population the sample is meant to represent, thereby allowing survey findings to generalize to the population of interest [8]. A poor coverage of the obtained sample may substantially bias survey findings which may severely affect their external validity [7, 8]. We previously reported on the representativeness of our sample in terms of age and socio-demographic characteristics [7]. As have others, we found the response rate (RR) to be higher in older men and in men living in rural areas while RRs were lower in higher income areas [7]. However, RRs may also depend on other aspects. Research has suggested that personality could be an important factor in the decision to take part in surveys [9]. At the same time, preferences for involvement in decision-making, which was the subject of our survey, could be hypothesized to be different among personality types [10–13] making a lack of representativeness of our sample particularly problematic. Thus, in addition to comparing respondents and non-respondents with respect to basic sociodemographic characteristics [8, 14, 15] it seems reasonable to consider also sample representativeness in regard to personality characteristics and baseline preferences [7]. *In this paper, through comparison with previously reported figures, we report on the representativeness of our survey sample in terms of personality and baseline preference characteristics.*

## Methods

### Setting and measures

Our survey illustrated various levels of patient involvement in healthcare decision-making through use of multiple versions of a case vignette [2]. We randomized participants into vignette versions and accompanying questions and measured their imagined satisfaction with the healthcare illustrated and participants’ desire to complain. The survey used Prostate-Specific Antigen (PSA) test for early prostate cancer (PCa) detection as a model for studying preferences for participation, as risks and benefits are delicately balanced and a choice for or against having the test may have far-reaching consequences. Based on existing recommendations regarding PSA testing, we chose men in the age span of 45–70 years [1, 16]. Measures comprised purpose-designed questions (e.g. socio-demographic characteristics) and standardized validated instruments, including personality measurement. We also aimed to achieve a ‘standardized’ baseline measure of participants’ preferences for involvement in healthcare decision-making holding everything else equal (meaning type of decision, the health care provider in question, etc.). Regarding participant personality, we used the BFI-10 (Big Five Inventory – 10) tool that has been previously used to investigate personality in studies of decision-making regarding PCa [17]. BFI-10 is a validated 10-item personality instrument that measures the Big Five personality dimensions *Extraversion*, *Agreeableness*, *Conscientiousness*, *Emotional Stability*, and *Openness* [18]. It was developed from the 44-item Big Five Inventory and designed for use in contexts with limited time available [18, 19]. We used the ‘*Control Preferences Scale*’ (CPS) for measuring baseline preferences for involvement in healthcare decision-making [20, 21]. The CPS is a validated instrument that has been previously used in studies on decision-making about cancer care [20]. Respondents are requested to mark one statement among five to describe the degree of control the respondent wants when health care decisions are made: A. “*I prefer to make the final selection about which treatment I will receive*,” B. “*I prefer to make the final selection of my treatment after seriously considering my doctor’s opinion*,” C. “*I prefer that my doctor and I share responsibility for deciding which treatment is best for me*,” D. “*I prefer that my doctor makes the final decision about which treatment, but seriously considers my opinion*,” or E. “*I prefer to leave all decisions regarding treatment to my doctor*” [21]. Through patient and public involvement in designing and fine-tuning the survey, we aimed to enhance survey acceptability and RRs in the invited population [2].

### Procedures

We used REDCap® for the survey and made invitations through the Danish authorities’ ‘digital mailbox’ that is a state authorized web platform for safe communication between citizens and the authorities. With due consideration to respecting people’s right to not participate in our survey, we chose to send out only one reminder after 14 days. In total, 6756 responded out of a sample of 24,000 male Danish Healthcare users randomly drawn by the Danish Health Data Authority amounting to a RR rate of 30 % [7]. We analyzed representativeness of the sample through comparisons of BFI-10 and CPS profiles of our respondents with BFI-10 and CPS figures reported in previous studies. Regarding BFI-10, we used survey data from the International Social Survey Program (ISSP, 2005) that includes BFI-10 data from different countries [22]. Data were retrieved from the Gesis database where open science collected data are made available to the scientific community (Gesis; Leibniz Institute for the Social Sciences) [23]. Research has previously suggested that personality profiles may vary among cultures and language areas [24]. Similarly, preferences for involvement in health care decision-making may differ among countries [25]. By way of example, findings from a study found differences between the views on patient involvement between United Kingdom and Sweden [26]. For comparisons, we selected 3 groups based on data from *a*) An ‘Anglophone’ group: US, United Kingdom, Canada, Australia, and New Zealand (US/UK/CA/AU/NZ); *b*) A ‘European-West Germanic group’: Germany, the Netherlands, Switzerland, and Belgium-Flemish Region (DE/NL/CH/BE-FL); and *c*) A ‘European-Scandinavian group’: Norway, Sweden, Finland, and (previous) Danish data (NO/SE/FI/DK). The aim of these choices was to both compare with countries assumed to be quite similar to Denmark, but on the other hand also to compare to large Western countries, to enable discussion of the external validity of our results outside Scandinavia as well. For control preference comparisons, repeated searches on PubMED revealed that scarce research data exist regarding CPS profiles in non-patient populations. We therefore used data from previously published studies deemed relevant from a cancer care perspective. We chose comparison data sets from studies, available from any one of the countries mentioned above (groups *a*, *b*, and *c*) from 2005 and onwards, and preferably concerning men and PCa. Three studies met the requirements [27–29].

### Statistics

To study representativeness, we compared our sample with the reference samples by reporting counts and proportions of each answer category, and testing distributions by chi-squared tests, respectively, or Fisher’s exact test in case of cell counts below five. We report numerical age with means and standard deviations compared by Wilcoxon rank sum tests. Stata version 16 (Stata-Corp, LP, College Station, TX) was used for the analysis and *p*-values below 0.05 were considered statistically significant.

## Results

### Comparisons of personality characteristics in sample and previous datasets

Table 1 compares the sociodemographic characteristics of our sample to group *a*-*c*. Bearing in mind the relatively large sample sizes, we established a statistically significant difference between socio-demographic characteristics of our survey sample and group a-c datasets used for comparison. More importantly, however, despite a slightly higher mean age of our sample, the characteristics of our sample fell within the range of the other aggregated data sets, as our estimates lie between those of the lowest and highest comparison groups. Besides, sample characteristics (marital status and working status compositions) bore closest resemblance to data from English speaking countries (Group *a*).

**Table 1:** Sample characteristics and norm data characteristics

	Sample (*n* = 6756)	Group *a* US/UK/CA/AU/NZ (*n* = 1512) (*)	Group *b* DE/NL/CH/BE-FL (*n* = 1136) (**)	Group *c* NO/SE/FI/DK (*n* = 1313) (***)
**Age** (mean, SD)	59.1 (7.3)	56.5 (7.3)	56.7 (7.4)	56.1 (6.9)
*P*-value		< 0.0001	< 0.0001	< 0.0001
**Marital status**				
Living together	5370 (79.5%)	1170(79.2%)	934 (83.6%)	1091 (85.0%)
Single *or* partner, not living together	1386 (20.5%)	308 (20.8%)	183 (16.4%)	193 (15.1%)
P-value		0.78	0.0014	< 0.0001
**Affiliation with labor market**				
Student/ …	11 (0.2%)	3 (0.2%)	0	5 (0.4%)
Working/ …	4445 (65.8%)	1043 (69.4%)	677 (60.6%)	908 (71.8%)
Unemployed/Retired	2300 (34.0%)	456 (31.4%)	440 (39.4%)	352 (27.9%)
*P*-value		0.023	0.0013	< 0.0001

*) Missing: 34 (marital status), 10 (affiliation with labor market), **)Missing: 19 (marital status), 19 (affiliation with labor market), ***)Missing: 29 (marital status), 48 (affiliation with labor market)

In Table 2, we compare personality characteristics of our sample in terms of the BFI-10 measure to the personality characteristics of group *a*-*c*. For distinct item ratings, please refer to Additional file 1. BFI factor ‘Agreeableness’ is determined from items 2 and 7 (reversed) while factor ‘Conscientiousness’ is derived from item 3 (reversed) and item 8. ‘Extraversion’ is determined from item 1 (reversed) and item 5, ‘Neuroticism’ is determined from item 4 (reversed) and item 9, and ‘Openness’ is determined from item 5 (reversed) and item 10 [18].

**Table 2:** Personality characteristics in sample and norm data

	Sample	Group *a* US/UK/CA/AU/NZ (mean (SD))	*P*-value	Group *b* DE/NL/ CH BE-FL (mean (SD))	*P*-value	Group *c* NO/SE/FI/DK (mean (SD))	*P*-value
**N**	6756	1512		1136		1313	
**BFI Factors**							
Agreeableness	4.68 (1.22)	4.61 (1.33)	0.095	4.80 (1.41)	0.040	4.43 (1.47)	0.006
Conscientiousness	4.18 (1.33)	3.49 (1.23)	< 0.001	3.57 (1.25)	< 0.001	3.26 (1.43)	< 0.001
Extraversion	4.71 (1.68)	5.62 (1.68)	< 0.001	5.23 (1.57)	< 0.001	4.41 (1.99)	< 0.001
Neuroticism	7.32 (1.59)	7.36 (1.67)	0.578	7.24 (1.58)	0.008	7.94 (1.87)	< 0.001
Openness	6.13 (1.65)	5.24 (1.33)	< 0.001	5.67 (1.30)	< 0.001	5.72 (1.40)	< 0.001
**How well do the following statements describe your personality?**							
**-** is reserved (1)	3.51 (1.04)	2.67 (1.10)	< 0.001	2.94 (1.09)	< 0.001	3.74 (1.27)	< 0.001
**-** is generally trusting (2)	2.01 (0.66)	1.91 (0.76)	< 0.001	2.30 (0.93)	< 0.001	1.73 (0.80)	< 0.001
-tends to be lazy (3)	3.86 (0.95)	4.13 (0.94)	< 0.001	4.07 (0.98)	< 0.001	4.15 (1.19)	< 0.001
**-**is relaxed, handles stress well (4)	2.32 (0.89)	2.30 (0.97)	0.472	2.41 (0.94)	< 0.001	2.11 (1.03)	< 0.001
**-**has few artistic interests (5)	2.96 (1.15)	2.77 (1.15)	< 0.001	2.64 (1.16)	< 0.001	2.26 (1.28)	< 0.001
**-**is outgoing, sociable (6)	2.22 (0.93)	2.28 (0.98)	0.077	2.17 (0.85)	0.574	2.15 (1.06)	0.034
**-**tends to find fault with others (7)	3.33 (0.94)	3.31 (0.98)	0.651	3.50 (0.95)	< 0.001	3.29 (1.18)	0.407
**-**does a thorough job (8)	2.04 (0.71)	1.62 (0.58)	< 0.001	1.64 (0.60)	< 0.001	1.40 (0.61)	< 0.001
**-**gets nervous easily (9)	3.63 (0.97)	3.65 (1.06)	0.345	3.65 (0.99)	0.884	4.06 (1.19)	< 0.001
**-**has an active imagination (10)	3.10 (1.09)	2.00 (0.85)	< 0.001	2.32 (0.92)	< 0.001	1.95 (0.93)	< 0.001

For three (and four for group c) out of five personality factors, there were significant differences between the study sample and the general population groups. However, factor mean scores of our sample fell within the range of group *a*-*c* in three out of five BFI factors. Again, if anything, the profile scores of our sample mostly resembled the profile of comparison group *a* (English speaking countries).

### Comparisons of baseline control preferences in sample and previous studies

Table 3 shows control preferences in the sample as well as comparisons made with CPS figures published in previous studies [27–29]. In concordance with the presentation of data in the studies compared to, we collapsed the original five categories of the CPS into only three (that is, ‘*active role*’, ‘*collaborative role*’, and ‘*passive role*’). Regarding the CPS profile of our sample, it mostly fell within the range of previously reported profiles.

**Table 3:** CPS characteristics in sample and comparison with previous studies

CPS	Sample (%)	Hack [27]	Noguera [28]	Yennurajalingam et al. [29]
Year	2019	2007	2014	2018
Sample size	6756	425	387	199
Country	Denmark	Canada	US	US
Population	Men aged 45–70 years	Men diagnosed with PCa	Patients with advanced cancer	Patients with advanced cancer
Age	59 (mean)	67 (mean)	58 (mean)	56 (median)
1 & 2 total (‘*Active role*’)	34.9% (2358)	30.6 (130)	31.2% (119)	43.2% (86)
3 (‘*Collaborative role*’)	41.6% (2810)	49.2 (209)	47.6% (182)	41.2% (82)
4 & 5 total (‘*Passive role*’)	23.5% (1588)	20.2 (86)	21.2% (81)	16.6% (33)
*P*-value		0.009	0.066	0.021

## Discussion

Using a state authorized web-based platform to distribute survey invitations provides an opportunity to rapidly get access to great numbers of potential respondents while reducing research costs [6]. The approach, however, can be challenged by issues relating to achieving a representative sample. In the invitation letter for our survey using the so-called digital mailbox, men were encouraged to participate to acquire knowledge about health care users’ preferences regarding participation in decisions about the medical care they receive. Unfortunately, however, only a minority chose to utilize this opportunity to have their voice heard, raising the question whether those who decided to respond are representative of our target population. Uneven representation of groups with different opinions regarding patient involvement could potentially introduce a significant non-responder bias requiring statistical adjustment be considered. We therefore previously reported on the representativeness of our sample in terms of socio-demographic characteristics and overall found our sample to fairly well represent the population of Danish adult men [7]. However, RRs were statistically significantly higher among older men and among men living outside the capital region but lower in high-income areas [7]. We finally concluded that our socio-demographic comparisons needed to be supplemented with studies of the representativeness regarding *personality* characteristics and respondents’ general view regarding patient involvement. In this paper, we compared the personality and control preference characteristics of our sample to previously collected international datasets. Despite minor variations, we found our sample to chiefly resemble international data. Below, we discuss findings in the context of the research literature.

### Sample personality characteristics

On three scales (agreeableness, extraversion, and neuroticism scales) out of five, rating estimates were within the range of our comparison groups (*a*-*c*). Regarding item ratings, our estimates fell within the range of comparison group averages in five items (reserved, trusting, relaxed, outgoing, fault finding) out of ten. Another five item profiles of our sample, especially relating to ‘Conscientiousness’ and ‘Openness’ scales, fell outside previously published figures. At least in part, our data may reflect the fact that population personality figures may not be constant over time and may have changed since ISSP data collections from 2005 [30]. It has been noted that the stability implied by the notion of ‘personality’ pertains to an individual life span and therefore does not preclude generational changes in personality trait distributions [30]. Correspondingly, previous cohort studies agreed that, e.g., ‘Conscientiousness’ ratings seem to increase over time [30, 31]. In spite of everything, the rating estimates in our sample mostly resembled those of the English-speaking countries rather than e.g. the Scandinavian countries group as a whole. The reason for this apparently greater similarity with English speaking countries remains unclear. It might reflect a greater similarity with English speaking countries but could also result from variation in the design of surveys and samples included in the Gesis data repository. In this regard, our comparisons in Table 1 suggest that sample compositions in terms of *socio-demographic characteristics* differ amongst groups and that sample differences may be smallest between our sample and the group of English speaking countries. In other words, regarding the personality and decision preference measures under study, our sample seems more similar to samples from English speaking countries than to, e.g., previously reported Scandinavian samples. Among others, similarities were clear regarding the trust item (and associated ‘agreeableness’ factor) that may be a particularly important aspect of personality, when considering healthcare communication and decision-making [32]. Physicians’ communication has an important impact on patients’ trust and trust generally is a crucial element of the healthcare provider-patient relationship. Correspondingly, trust may be particularly important when patients are in an exposed situation and, e.g., confront a potentially life-threatening illness such as cancer and therefore need to rely strongly on their care providers [33–35].

### Control preferences

Regarding control preference profiles, our sample not only displays a distribution that pretty nicely reflects the (‘bell’) distribution that has been repeatedly reported in the literature [36–38], but also specifically resemble CPS profiles reported in studies on men’s’ preferences for involvement in cancer care decisions. In this regard, it is remarkable that, if comparing to Degner and Sloan’s 27 year old data from the ‘pre-patient-involvement-era’, it appears that control preference figures have generally changed in favor of a more collaborative or active role [38, 39]. Still, it must be remembered that most other studies have been conducted in patient samples. As such, Degner and Sloan’s original study is among the few studies including also non-patients [20]. On the other hand, it probably could be claimed that similarity of our sample CPS figures with the control preferences of real life patients would be of great importance. Hence, it seems as if the preferences for involvement in decision-making of our sample is rather similar to patient preferences found in real life settings [27–29].

### Consideration regarding limitations and strengths

This leads to considering limitations and strengths of our study in more detail. It would be undesirable to conclude from a survey with a 30 % response rate that the population generally wants to participate in health care decision making if those 70 % of the population not wanting to participate in the survey are people who would generally abstain from any participation in health care decision making [7, 40]. Similarly, ‘norm data’ may not necessarily always be representative of the relevant population. For example, to the authors’ knowledge, it remains unclear to what extent, e.g., ISSP 2005 datasets were representative themselves of the countries studied. Findings from previous research suggested that study participation may itself depend on *personality* factors [41, 42]. For example, students in a survey were found to be more likely to be socially engaged (‘investigative’) personality types while they were less likely to be ‘enterprising’ or ‘artistic’ types [43]. In this regard, Holland personality typology was used with ‘enterprising’ or ‘artistic’ types partially correlating with Big Five’s *extraversion* and *openness* factors [9, 43]. In other words, individuals who score high on extroversion and openness seem less willing to participate in research studies [9, 41]. On the other hand, for example openness may be related to a lower probability of *quitting* a survey following recruitment and the relationship between personality and survey participation thus may not be that clear [42]. Correspondingly, little is known about the *association between decision-making preferences and survey participation*, and it is still possible that those not participating in our survey may have dissimilar personalities and control preferences. This hardly can be ruled out without just comparing to responders in another survey. Hence, strictly speaking, we have demonstrated that to a considerable extent, our sample seems comparable to international survey samples and that it is therefore likely that our forthcoming survey findings regarding preferences for participation in decision-making can be replicated abroad. Specifically regarding our comparison groups, as we found no commonly accepted categories in which culturally the BFI scores are distinctly different, we chose to group countries on our own although with reference to some relatively well known ‘categories’ (Western world English speaking countries, Scandinavia, and European West Germanic area). The latter categorization also explains the specific selection of countries.

## Conclusion

Achieving reasonable representativeness of the population under study in survey research is highly desirable to allow for drawing inferences from survey findings. Following a survey on men’s view on patient involvement in health care decision-making, we wanted to establish our sample’s representativeness. With particular focus on personality and baseline involvement preferences, we studied whether those who responded rated similarly to those participating in previous surveys. Despite some variation, we found our sample to very well resemble pre-existing international data which is important when interpreting findings from further analyses of our survey responses and making generalizations to an international context.

## Supplementary information

**Additional file 1. **BFI personality trait ratings in sample and norm data.

## Data Availability

The data that support the findings of this study are available from Open Patient Exploratory Network (OPEN) but restrictions apply to the availability of these data, which were used under license for the current study, and so are not publicly available. Data are however available from the authors upon reasonable request and with permission of the Danish Data Protection Agency.

## Abbreviations

AU: Australia
BE-FL: Belgium-Flemish Region
BFI: Big Five Inventory
CA: Canada
CH: Switzerland
CPS: Control Preferences Scale
DE: Germany
DK: Denmark
FI: Finland
Gesis: Gesellschaft Sozialwissenschaftlicher Infrastruktureinrichtungen
ISSP: International Social Survey Program
NL: the Netherlands
NO: Norway
NZ: New Zealand
OR: Odds ratio
PCa: Prostate cancer
PSA: Prostate-specific antigen
REDCap: Research electronic data capture
RR: Response rate
SE: Sweden
UK: United Kingdom
US: United States
