# Supplementary material for: Representativeness of personality and involvement preferences in a web-based survey on healthcare decision-making
Source: BMC Health Serv Res. 2020 Sep 10;20:851. doi: 10.1186/s12913-020-05717-1 (PMC7488239; doi:10.1186/s12913-020-05717-1)
Supplement: Supplementary file 1 — Additional file 1. BFI personality trait ratings in sample and norm data. [file 12913_2020_5717_MOESM1_ESM.docx]

**APPENDIX 1 BFI personality trait ratings in sample and norm data**

| **How well do the following statements describe your personality?** | Sample | Norm data  US/UK/CA/AU/NZ | P-value | Norm data  DE-W(DE E/NL/ CH BE-FL | P-value | Norm data  NO/SE/FI/DK | P-value |
| --- | --- | --- | --- | --- | --- | --- | --- |
| **-** is reserved (1) |  |  |  |  |  |  |  |
| rate 1 | 130 (1.92%) | 57 (9.61%) | <0.001 | 73 (6.68%) | <0.001 | 17 (4.63%) | <0.001 |
| rate 2 | 1506 (22.29%) | 280 (47.22%) |  | 375 (34.34%) |  | 64 (17.44%) |  |
| rate 3 | 862 (12.76%) | 98 (16.53%) |  | 270 (24.73%) |  | 63 (17.17%) |  |
| rate 4 | 3316 (49.08%) | 119 (20.07%) |  | 291 (26.65%) |  | 77 (20.98%) |  |
| rate 5 | 942 (13.94%) | 39 (6.58%) |  | 83 (7.60%) |  | 146 (39.78%) |  |
| **-** is generally trusting (2) |  |  |  |  |  |  |  |
| rate 1 | 1040 (15.39%) | 158 (26.20%) | <0.001 | 162 (14.62%) | <0.001 | 165 (43.65%) | <0.001 |
| rate 2 | 4971 (73.58%) | 378 (62.69%) |  | 622 (56.14%) |  | 167 (44.18%) |  |
| rate 3 | 402 (5.95%) | 34 (5.64%) |  | 181 (16.34%) |  | 32 (8.47%) |  |
| rate 4 | 321 (4.75%) | 28 (4.64%) |  | 113 (10.20%) |  | 11 (2.91%) |  |
| rate 5 | 22 (0.33%) | 5 (0.83%) |  | 30 (2.71%) |  | 3 (0.79%) |  |
| - tends to be lazy (3) |  |  |  |  |  |  |  |
| rate 1 | 74 (1.10%) | 16 (2.68%) | <0.001 | 21 (1.89%) | <0.001 | 13 (3.49%) | <0.001 |
| rate 2 | 821 (12.15%) | 29 (4.86%) |  | 88 (7.91%) |  | 38 (10.19%) |  |
| rate 3 | 692 (10.24%) | 47 (7.87%) |  | 111 (9.98%) |  | 48 (12.87%) |  |
| rate 4 | 3560 (52.69%) | 275 (46.06%) |  | 465 (41.82%) |  | 55 (14.75%) |  |
| rate 5 | 1609 (23.82%) | 230 (38.53%) |  | 427 (38.40%) |  | 219 (58.71%) |  |
| **-** is relaxed, handles stress well (4) |  |  |  |  |  |  |  |
| rate 1 | 873 (12.92%) | 107 (17.83%) | 0.001 | 157 (14.17%) | <0.001 | 125 (33.24%) | <0.001 |
| rate 2 | 3862 (57.16%) | 301 (50.17%) |  | 510 (46.03%) |  | 137 (36.44%) |  |
| rate 3 | 1134 (16.79%) | 109 (18.17%) |  | 286 (25.81%) |  | 67 (17.82%) |  |
| rate 4 | 785 (11.62%) | 69 (11.50%) |  | 136 (12.27%) |  | 42 (11.17%) |  |
| rate 5 | 102 (1.51%) | 14 (2.33%) |  | 19 (1.71%) |  | 5 (1.33%) |  |
| **-** has few artistic interests (5) |  |  |  |  |  |  |  |
| rate 1 | 533 (7.89%) | 83 (13.86%) | <0.001 | 199 (18.03%) | <0.001 | 153 (40.91%) | <0.001 |
| rate 2 | 2440 (36.12%) | 201 (33.56%) |  | 357 (32.34%) |  | 70 (18.72%) |  |
| rate 3 | 1093 (16.18%) | 119 (19.87%) |  | 248 (22.46%) |  | 70 (18.72%) |  |
| rate 4 | 2121 (31.39%) | 163 (27.21%) |  | 242 (21.92%) |  | 64 (17.11%) |  |
| rate 5 | 569 (8.42%) | 33 (5.51%) |  | 58 (5.25%) |  | 17 (4.55%) |  |
| **-** is outgoing, sociable (6) |  |  |  |  |  |  |  |
| rate 1 | 1308 (19.36%) | 122 (20.40%) | <0.001 | 204 (18.43%) | <0.001 | 122 (32.11%) | <0.001 |
| rate 2 | 3639 (53.86%) | 276 (46.15%) |  | 613 (55.37%) |  | 137 (36.05%) |  |
| rate 3 | 918 (13.59%) | 117 (19.57%) |  | 200 (18.07%) |  | 74 (19.47%) |  |
| rate 4 | 829 (12.27%) | 74 (12.37%) |  | 75 (6.78%) |  | 37 (9.74%) |  |
| rate 5 | 62 (0.92%) | 9 (1.51%) |  | 15 (1.36%) |  | 10 (2.63%) |  |
| **-** tends to find fault with others (7) |  |  |  |  |  |  |  |
| rate 1 | 110 (1.63%) | 18 (3.02%) | 0.017 | 27 (2.44%) | <0.001 | 18 (4.88%) | <0.001 |
| rate 2 | 1506 (22.29%) | 125 (20.97%) |  | 161 (14.56%) |  | 91 (24.66%) |  |
| rate 3 | 1588 (23.51%) | 153 (25.67%) |  | 269 (24.32%) |  | 101 (27.37%) |  |
| rate 4 | 3157 (46.73%) | 255 (42.79%) |  | 527 (47.65%) |  | 84 (22.76%) |  |
| rate 5 | 395 (5.85%) | 45 (7.55%) |  | 122 (11.03%) |  | 75 (20.33%) |  |
| **-** does a thorough job (8) |  |  |  |  |  |  |  |
| rate 1 | 1211 (17.92%) | 252 (42.00%) | <0.001 | 457 (41.25%) | <0.001 | 247 (65.00%) | <0.001 |
| rate 2 | 4369 (64.67%) | 328 (54.67%) |  | 606 (54.69%) |  | 118 (31.05%) |  |
| rate 3 | 869 (12.86%) | 15 (2.50%) |  | 37 (3.34%) |  | 12 (3.16%) |  |
| rate 4 | 291 (4.31%) | 5 (0.83%) |  | 4 (0.36%) |  | 2 (0.53%) |  |
| rate 5 | 16 (0.24%) | 0 (0.00%) |  | 4 (0.36%) |  | 1 (0.26%) |  |
| **-** gets nervous easily (9) |  |  |  |  |  |  |  |
| rate 1 | 143 (2.12%) | 19 (3.16%) | 0.002 | 16 (1.44%) | <0.001 | 15 (4.00%) | <0.001 |
| rate 2 | 1008 (14.92%) | 92 (15.31%) |  | 159 (14.31%) |  | 35 (9.33%) |  |
| rate 3 | 1005 (14.88%) | 88 (14.64%) |  | 238 (21.42%) |  | 60 (16.00%) |  |
| rate 4 | 3626 (53.67%) | 284 (47.25%) |  | 483 (43.47%) |  | 69 (18.40%) |  |
| rate 5 | 974 (14.42%) | 118 (19.63%) |  | 215 (19.35%) |  | 196 (52.27%) |  |
| **-** has an active imagination (10) |  |  |  |  |  |  |  |
| rate 1 | 407 (6.02%) | 170 (28.43%) | <0.001 | 181 (16.44%) | <0.001 | 136 (36.86%) | <0.001 |
| rate 2 | 1984 (29.37%) | 298 (49.83%) |  | 529 (48.05%) |  | 141 (38.21%) |  |
| rate 3 | 1438 (21.28%) | 89 (14.88%) |  | 264 (23.98%) |  | 69 (18.70%) |  |
| rate 4 | 2400 (35.52%) | 40 (6.69%) |  | 107 (9.72%) |  | 19 (5.15%) |  |
| rate 5 | 527 (7.80%) | 1 (0.17%) |  | 20 (1.82%) |  | 4 (1.08%) |  |
